# Effects of Chronic Administration of Green Tea Ethanol Extract on Sleep Architecture in Mice: A Comparative Study with a Representative Stimulant Caffeine

**DOI:** 10.3390/nu15041042

**Published:** 2023-02-20

**Authors:** Duhyeon Kim, Seonghui Kim, Minseok Yoon, Min Young Um, Suengmok Cho

**Affiliations:** 1 Department of Food Science and Technology, Institute of Food Science, Pukyong National University, Busan 48513, Republic of Korea; 2 Research Division of Food Functionality, Korea Food Research Institute, Wanju 55365, Republic of Korea

**Keywords:** arousal-inducing effect, caffeine, *Camellia sinensis* L., green tea ethanol extract, polysomnographic recording

## Abstract

Wakefulness is defined as a state in which individuals can react to a change in situations. The number of people staying awake and compensating for lack of sleep has increased in recent years. Caffeine, a representative stimulant, is the most extensively consumed compound globally and is mainly consumed through coffee. Although green tea (*Camellia sinensis* L.) contains high caffeine content like coffee, its arousal-inducing effects have not yet been studied. In the present study, we aimed to identify the arousal-inducing effect of GT during a chronic administration period (three weeks) using analysis of sleep architecture. Treatment with GT (1500 mg/kg) significantly elevated the sleep latency and wakefulness throughout the treatment period, and chronic administration of GT consistently maintained an increase in wakefulness for up to 3 h. During the treatment period, the arousal-inducing effect of GT (1500 mg/kg) occurred without any change in the tolerance phenomenon or withdrawal symptoms, similar to that observed with caffeine (25 mg/kg). GT (1500 mg/kg) containing 95.6 mg/kg of caffeine did not produce a better arousal-inducing effect than caffeine at 25 mg/kg. These results indicate that the arousal-inducing effect of GT persisted for three weeks without adverse effects and that GT can control the arousal-inducing effects of caffeine due to the hypnotic effects of its other constituents.

## 1. Introduction

Wakefulness is usually defined as a state in which individuals can physically react to a change in situations [1]. Additionally, the physiological part of the brain is activated by fast, low-amplitude, and unsettled oscillations during wakefulness [2]. Normal wakefulness is involved in neuronal activity of ascending arousal systems, including monoaminergic neurons, the posterior hypothalamus, cholinergic neurons, and orexin [3]. Historically, there has been a demand to stay awake and compensate for poor sleep in conditions such as insomnia, which is the difficulty in initiating and retaining sleep, and is mainly associated with daytime sleepiness [4]. As a result, excessive daytime sleepiness attenuates quality of life.

To avoid drowsiness and poor concentration at the workplace, most people consume tea, coffee, or energy drinks. A previous study confirmed that about 85% of the U.S. population drinks one or more caffeinated beverages daily [5]. The majority of these stimulants that assist in wakefulness contain caffeine [6]. Caffeine, the most extensively used psychoactive substance, causes arousal effects by blocking adenosine A_2A_ receptors [7]. Moreover, consumption of caffeine can lead to improved wakefulness and decreased fatigue [8]. Various studies related to the arousal effects of caffeine in humans and animals have reported prolonged sleep latency and a state of wakefulness [9,10,11,12]. A recent study reported that coffee is the main source of caffeine, accounting for approximately 80% of total intake of caffeine in adults [13]. 

In addition to coffee, green tea is another predominant source of caffeine intake. Green tea (*Camellia sinensis* L.), originating from China and Southeast Asia, has been used as a traditional herbal remedy and functional food for centuries [14,15] and provides antioxidant [16], antimicrobial [17], antitumor [18], and antibacterial [19] effects. Green tea is known to contain various bioactive compounds, such as caffeine, (−)-epigallocatechin-3-*O*-gallate (EGCG), quercetin, and L-theanine [20,21,22]. Among these compounds, a previous study reported high caffeine content of dried green tea leaves ranging from 33.5 ± 6.6–49.2 ± 3.6 mg/g [23]. Despite the reported high caffeine content in green tea, reports on the arousal-inducing effects in comparison to coffee remain lacking. Moreover, in a previous study by Lin et al. [24], green tea ethanolic extract showed higher contents of biologically active compounds than that in the aqueous extract. Hu et al. [25] confirmed the caffeine content of green tea extracted with 70% ethanol to be three times greater than that of water. In addition, several studies have demonstrated that, compared to the aqueous extract of green tea, ethanol has better bioactivity.

Considering the higher biological activities of green tea ethanol extract (GT) compared with water, it is evident that GT can induce arousal effects. Thus, we aimed to investigate whether GT maintained arousal-inducing effects during the three-week study period. In the present study, we analyzed the arousal-inducing effect of GT using polysomnographic recordings in C57BL/6N mice and determined whether GT caused a tolerance phenomenon over the treatment period or any adverse effects after treatment completion.

## 2. Materials and Methods

### 2.1. Materials

Pentobarbital was used as an anesthetic drug for mouse surgery and was purchased from Hanlim Pharm. Co., Ltd. (Seoul, Republic of Korea). Caffeine, which was used as a comparative drug, was purchased from Sigma-Aldrich, Inc. (St. Louis, MO, USA). To analyze caffeine content of GT, acetonitrile and methanol were purchased from Burdick & Jackson (Muskegon, MI, USA). All chemical reagents used in the experiments were of the highest analytical grade.

### 2.2. Preparation of GT

Dried green tea leaves were obtained from the Hanyakjae Market (Seoul, Republic of Korea). Green tea leaves were extracted with 60% (*v*/*v*) ethanol–water solution at 50 °C for 30 min. After concentrating extracts using a rotary evaporator, the extracts were powdered through lyophilization.

### 2.3. Animals

The use of experimental animals was approved by the Animal Care and Use Committee of the Korea Food Research Institute (approval code: KFRI-M-21001; approval date: 8 January 2021) and the Institutional Animal Care and Use Committee of Pukyong National University (approval code: PKNUIACUC-2022-20; approval date: 14 April 2022). C57BL/6N mice from KOATECH, Inc. (Pyeongtaek, Republic of Korea) were used at 11 weeks of age. All animals were raised in an isolated and sound-proofed recording room under a constant indoor temperature of 23 ± 0.5 °C and a relative humidity of 55 ± 2%. Before surgery for polysomnographic recordings, mice were acclimatized for a week. The animals were handled on a 12 h light/dark cycle (lights on at 09:00 h) and were provided a free supply of chow and water in individual cages. A total of 8 mice were used in this study.

### 2.4. HPLC Analysis Conditions of GT

#### 2.4.1. Preparation of Sample Solution

A stock solution of caffeine, which was used as a reference standard, was dissolved in 50% methanol. The caffeine standard was diluted to seven concentrations (1, 5, 10, 25, 50, 100, and 200 μg/mL). The correlation coefficient (*R*^2^) values were found to be 0.9999. The GT sample solution was sonicated and filtered through a PVDF syringe filter (0.45 μm). After preparation of the GT sample, it was used as a sample solution for HPLC analysis. The concentration of samples was 1 mg/mL in 50% methanol.

#### 2.4.2. HPLC Analysis of Caffeine and GT

High-performance liquid chromatography (HPLC) analysis was conducted using a Hitachi CM-5000 series HPLC system (Hitachi Ltd., Tokyo, Japan consisting of a CM-5110 pump, a CM-5280 autosampler, a CM-5410 UV detector, a CM-5310 column oven, and an organizer. A CAPCELL PAK C18 MG column (250 mm × 4.6 mm, particle size: 5 μm, OSAKA SODA, Tokyo, Japan) was monitored at 278 nm and a flow rate of 1.0 mL/min. The column oven temperature was set to 40 °C. The mobile phase was composed of 0.1% acetic acid in water and acetonitrile (80:20, *v*/*v*). The injection volume of samples was 10 µL.

### 2.5. Analysis of Sleep Architecture and Sleep–Wake Profile

#### 2.5.1. Surgical Procedure

Polysomnographic analysis of C57BL/6N mice was conducted using the method described by UM et al. [26]. A single head mount (Pinnacle Technology, Inc., Lawrence, KS, USA) containing electroencephalogram (EEG) and electromyogram (EMG) electrodes was affixed on the skull of mice to record polysomnographic signals. After the pentobarbital (50 mg/kg) injection was administered, the hair on the neck of mice was shaved, the skin was disinfected with ethanol, and the head mount was positioned anteriorly 3.0 mm from the bregma. Four screws were inserted—two each on the front and back holes. Double-wire electrodes were implanted on both sides of the nuchal muscles for EMG recording. Dental cement was used to prevent separation of the head mount from the skull. Postoperatively, the mice were adapted in an environment to mimic the recording conditions for four days. 

#### 2.5.2. Pharmacological Treatment

**Figure 1:**
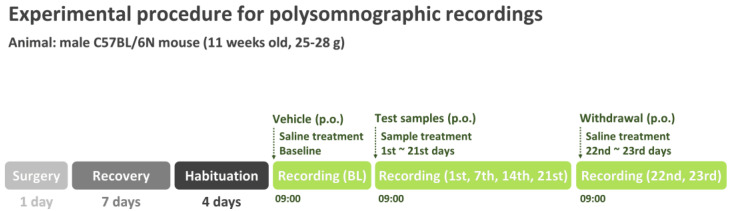
Experimental procedure involving chronic administration of GT for polysomnographic recordings. BL, baseline; p.o., per os administration.

The treatment procedure for analysis of sleep architecture was conducted according to Figure 1. Sterile saline including 5% Tween 80 solution was used to dissolve test samples. GT (1500 mg/kg) and caffeine (25 mg/kg) were administered orally to C57BL/6N mice (*n* = 7–8 per group) at 09:00 h daily for 21 consecutive days. EEG and EMG data were recorded at baseline (BL) and on the 1st, 7th, 14th, and 21st days, as well as over the withdrawal period (22nd and 23rd days).

#### 2.5.3. Electroencephalography and Electromyography Recordings

EEG and EMG recordings were carried out using a slip ring designed to allow for free behavioral movement of mice. To analyze the EEG and EMG signals, a PAL-8200 three-channel acquisition system (Pinnacle Technology Inc., Lawrence, KS, USA) was used at a sampling rate of 200 Hz. All signals were controlled under the conditions of amplifying action at 100-fold and low-pass filtering at 10 Hz. Monitoring was conducted for 48 h, and baseline data for control and sample groups were recorded for 24 h each.

#### 2.5.4. Sleep–Wake State Analysis 

The epoch was calculated as 10 s, and the vigilance states were divided into non-rapid-eye-movement sleep, rapid-eye-movement sleep (NREMS and REMS, respectively, and wakefulness using SleepSign software (Kissei Comtec, Nagano, Japan) [26]. After oral administration of test samples, sleep latency was defined as a duration lasting more than 12 epochs from the first NREMS. The classified sleep–wake stages were confirmed directly and corrected if misclassified. The delta activity with a range of 0.5–4 Hz was added during NREMS and calculated as a mean percentage of the corresponding delta power value of NREMS. The bouts in each stage were determined as consecutive epochs of more than 10 s.

### 2.6. Statistical Analysis

Statistical analyses were conducted using GraphPad Prism version 8.0 (GraphPad Software, Inc., San Diego, CA, USA). All data were presented as the mean ± standard error of the mean (SEM). To analyze multiple comparisons, data were expressed using one- and two-way analyses of variance following Dunnett’s test. A comparison between the two groups was conducted using a paired Student’s *t*-test. *p*-values less than 0.05 were considered significant for all statistics.

## 3. Results

### 3.1. Effects of Chronic Administration of GT on Sleep Latency and NREMS Amount

**Figure 2:**
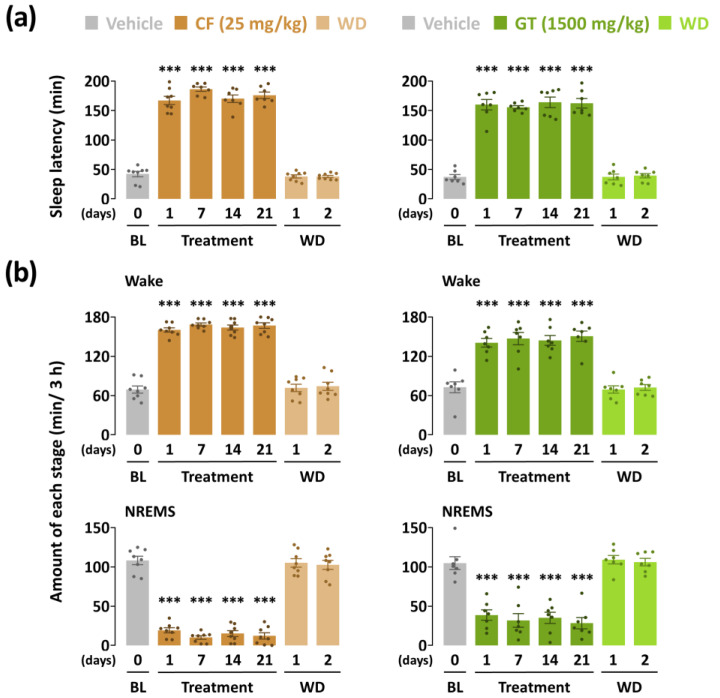
Effects of chronic administration of caffeine (25 mg/kg) and GT (1500 mg/kg) on (**a**) sleep latency and (**b**) amount of wakefulness and NREMS stages during the 3 h after administration. Grey bars illustrate the baseline day (vehicle). Brown and green bars illustrate caffeine and GT treatments, respectively. Light brown and green bars illustrate the withdrawal (WD) days after caffeine and GT treatments, respectively. Every column denotes the mean ± SEM (*n* = 7–8). *** *p* < 0.001 indicates significant differences compared to the vehicle (Dunnett’s test). BL, baseline; CF, caffeine; GT, green tea ethanol extract; NREMS, non-rapid-eye-movement sleep; Wake, wakefulness; SEM, standard error of the mean.

To evaluate the arousal-inducing effect of GT (1500 mg/kg), we used caffeine as a reference stimulant drug. Until sleep onset, the sleep latency of GT increased (*p* < 0.001) during the entire treatment period (Figure 2a). Sleep latency of the caffeine-administered group also increased significantly during the 21 days. As shown in Figure 2b, the total time was spent in wakefulness and NREMS stages during the first 3 h after the administration of GT and caffeine. However, both the GT- and caffeine-administered groups showed insignificant changes during the withdrawal period. GT at 1500 mg/kg maintained arousal-inducing effects 3 h after administration from the first to the last day. Wakefulness in the first and last days of GT treatment was 140.7 ± 6.6 and 150.7 ± 7.8 min, respectively. Compared to the baseline group, GT was statistically potentiated by a 1.9-fold (*p* < 0.001) and 2.1-fold (*p* < 0.001) increase, respectively. In the caffeine treatment group, the first-to-last-day findings illustrate a continuous elevation of sleep latency ranging from 2.3-fold (*p* < 0.001) to 2.4-fold (*p* < 0.001) compared to the baseline group. The NREMS levels of the two groups during the 3 h following administration presented a significant reduction (*p* < 0.001) throughout the treatment period. Insignificant changes were observed in the all experimental groups during the withdrawal days.

### 3.2. Effects of Chronic Administration of GT on Time-Course Changes in Sleep–Wake Stages

**Figure 3:**
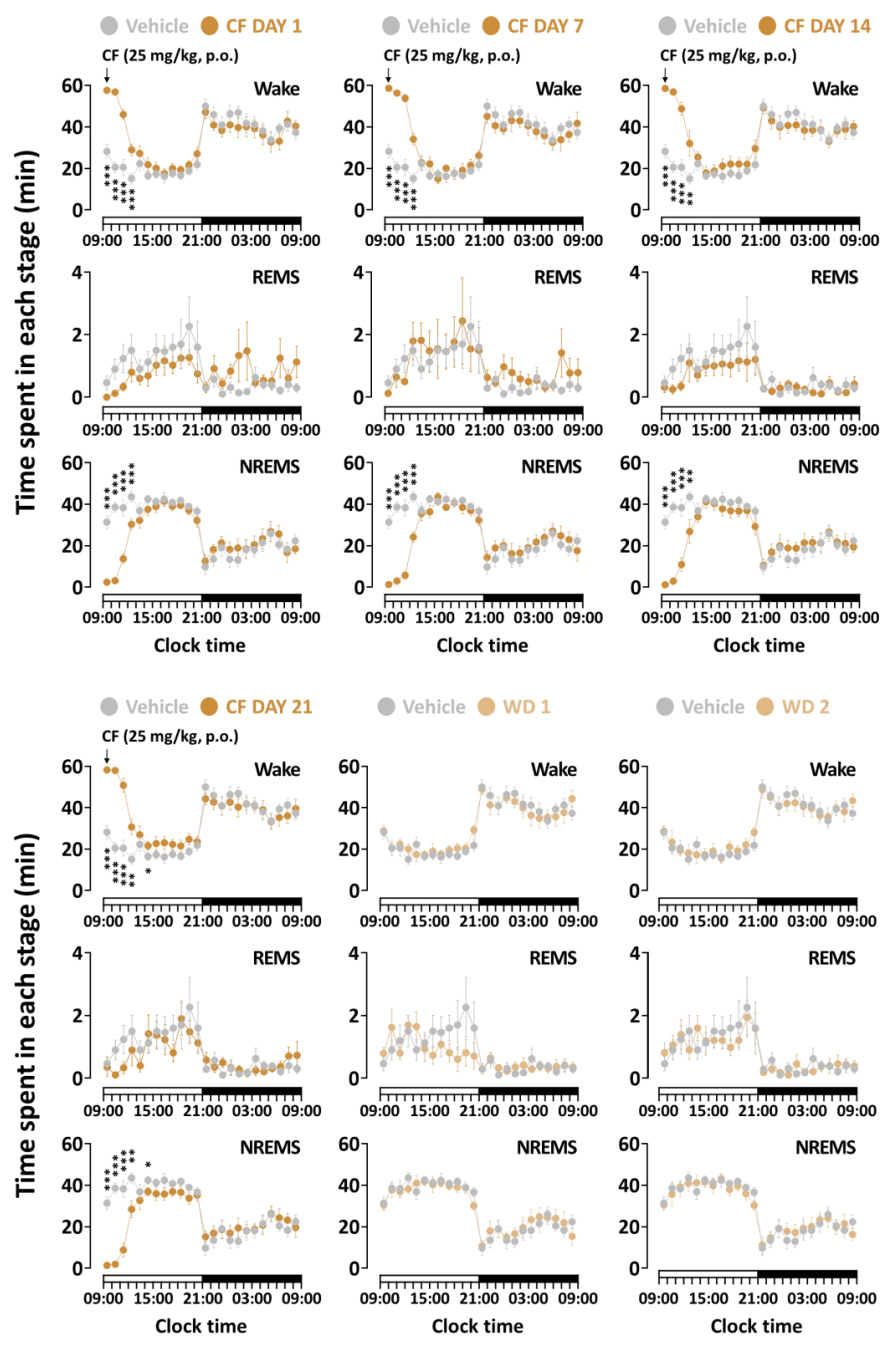
Time-course changes of caffeine (25 mg/kg) treatment during each stage over the whole administration period. Grey circles illustrate the baseline day (vehicle). Brown circles illustrate caffeine treatment. Light brown circles illustrate the withdrawal (WD) day during the end of caffeine treatment. Every circle denotes the hourly mean ± SEM (*n* = 7–8) in each stage. * *p* < 0.05, ** *p* < 0.01, and *** *p* < 0.001 indicate significant differences compared to the vehicle (Dunnett’s test). BL, baseline; CF, caffeine; NREMS, non-rapid-eye-movement sleep; REMS, rapid-eye-movement sleep; Wake, wakefulness; SEM, standard error of the mean.

**Figure 4:**
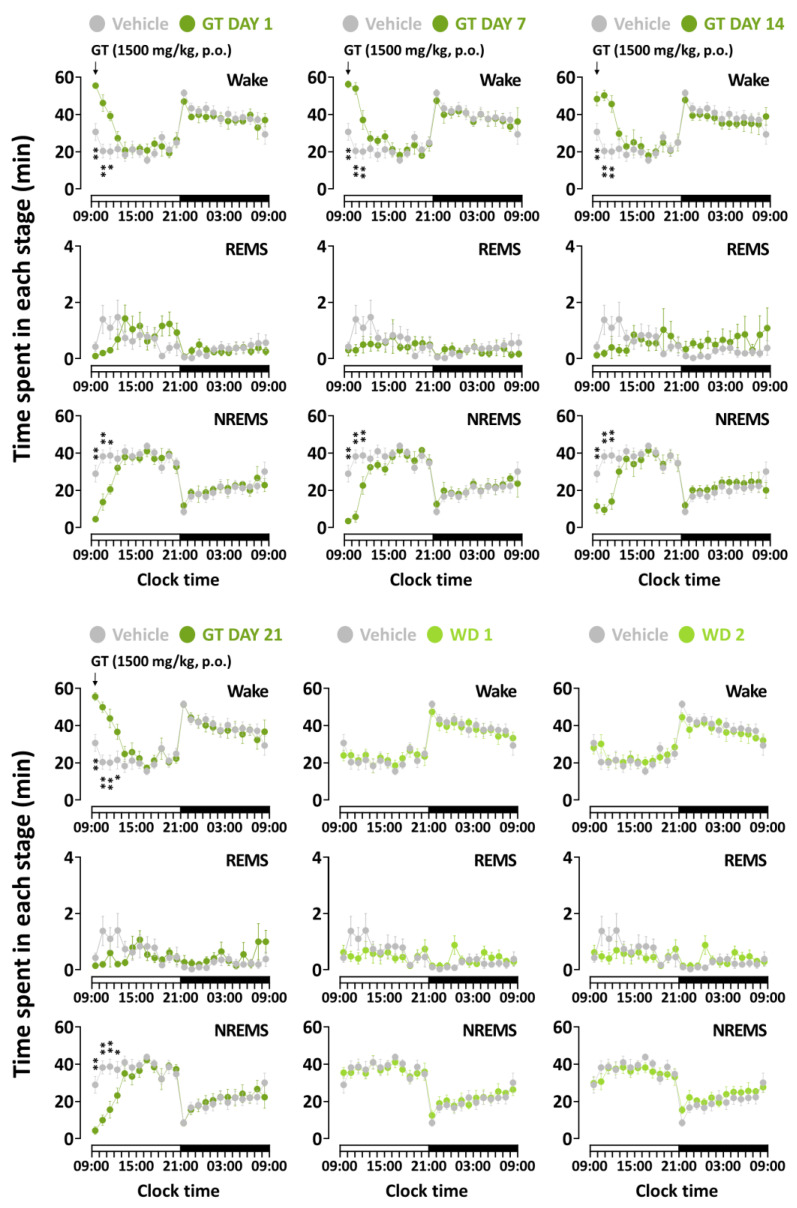
Time-course changes of GT (1500 mg/kg) treatment during each stage over the whole administration period. Grey circles indicate the baseline day (vehicle). Green circles illustrate GT treatment. Light green circles illustrate the withdrawal (WD) day during the end of GT treatment. Every circle denotes the hourly mean ± SEM (*n* = 7–8) in each stage. * *p* < 0.05, ** *p* < 0.01 indicate significant differences compared to the vehicle (Dunnett’s test). BL, baseline; GT, green tea ethanol extract; NREMS, non-rapid-eye-movement sleep; REMS, rapid-eye-movement sleep; Wake, wakefulness; SEM, standard error of the mean.

Figure 3 and Figure 4 present the time-course changes for 24 h of all treatment groups during the three weeks of the study. Caffeine at 25 mg/kg, which was used as a positive control, maintained an increase in wakefulness for more than 4 h from the first to the 14th day (Figure 2a). The changes on the last day showed a significant elevation of wakefulness at the 4th (1st to 3rd, *p* < 0.001; 4th, *p* < 0.01) and 6th (*p* < 0.05) time of administration. These values are in line with statistically similar changes in the NREMS amount. GT also presented arousal-inducing effects on wakefulness (Figure 2b). Compared to that of baseline, GT showed persistent effects 3 h after treatment during the three weeks. A significant change was observed in both wakefulness and the NREMS stage at during the 4th administration (1st to 3rd, *p* < 0.01; 4th, *p* < 0.05) on the 21st day of administration. When the sample treatment was stopped during the withdrawal period, both GT and caffeine led to effects with insignificant differences. These results imply that GT was effective only while it was administered to mice, similar to results observed with caffeine.

### 3.3. Effects of Chronic Administration of GT on Characteristics of Sleep–Wake Episodes

**Figure 5:**
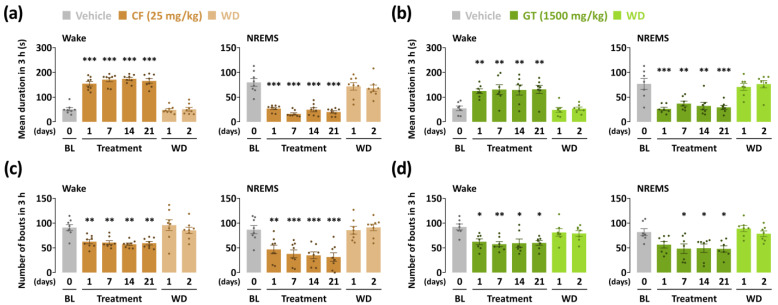
Features of sleep–wake bouts during the 3 h following sample treatment for the chronic administration period (21 days). (**a**) Changes in mean duration of wakefulness and NREMS after caffeine (25 mg/kg) treatment. (**b**) Changes in mean duration of wakefulness and NREMS after GT (1500 mg/kg) treatment. (**c**) The number of bouts of wakefulness and NREMS after caffeine treatment. (**d**) The number of bouts of wakefulness and NREMS after GT treatment. Grey bars illustrate the baseline day (vehicle). Brown and green bars illustrate days when caffeine and GT treatment were administered, respectively. Light brown and green bars illustrate withdrawal (WD) days after caffeine and GT treatments, respectively. Every column denotes the mean ± SEM (*n* = 7–8). * *p* < 0.05, ** *p* < 0.01, and *** *p* < 0.001, indicate significant differences compared to the vehicle (Dunnett’s test). CF, caffeine; GT, green tea ethanol extract; NREMS, non-rapid-eye-movement sleep; Wake, wakefulness; SEM, standard error of the mean.

The mean duration of wakefulness in the caffeine group significantly increased in the range of 3.0–3.4-fold (*p* < 0.001) for the three weeks compared with the vehicle group (Figure 5a). Although less than caffeine during the same period, GT also showed a significant increase in wakefulness, as reflected by an increase of 2.3-fold (*p* < 0.01) to 2.4-fold (*p* < 0.01) (Figure 5b). The number of bouts of caffeine administration showed continuous augmentation in both wakefulness and NREMS stages (Figure 5c). Although not as much as caffeine, GT also increased and decreased the wakefulness and NREMS bouts, respectively (Figure 5d).

**Figure 6:**
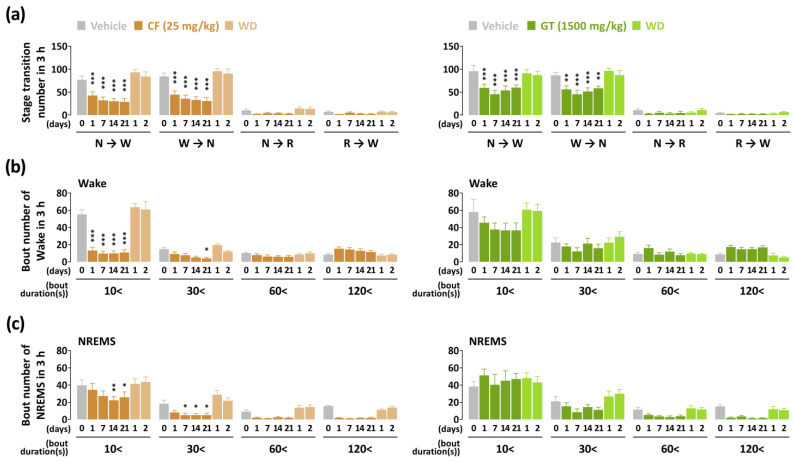
(**a**) Changes in stage transition number during the 3 h following caffeine (25 mg/kg) and GT (1500 mg/kg) treatments for the chronic administration period (21 days). Changes in the number of bouts of (**b**) wakefulness and (**c**) NREMS after caffeine and GT treatments during the chronic administration period (21 days). Grey bars illustrate the baseline day (vehicle). Brown and green bars illustrate caffeine and GT treatments, respectively. Light green and brown bars illustrate the withdrawal (WD) day after of GT and caffeine treatments, respectively. Every column denotes the mean ± SEM (*n* = 7–8). * *p* < 0.05, ** *p* < 0.01, and *** *p* < 0.001 indicate significant differences compared to the vehicle (Dunnett’s test). CF, caffeine; GT, green tea ethanol extract; NREMS, non-rapid-eye-movement sleep; Wake, wakefulness; SEM, standard error of the mean.

The stage transition number alone changed between the NREMS and wakefulness stages in the two samples (Figure 6a). The value of stage transition from NREMS to wakefulness decreased from 45% (1st day, *p* < 0.001) to 63% (21st day, *p* < 0.001) in the caffeine treatment group compared to the baseline group. GT treatment group from NREMS to wakefulness showed a significant decrease from 37% (7th day, *p* < 0.001) to 53% (21st day, *p* < 0.001). The number of stage transitions from wakefulness to NREMS declined, and the value was similar to that seen for the transition from NREMS to wakefulness in both groups. While administration of caffeine presented significant changes ranging from 10 to 30 s, GT did not show any change in wakefulness and NREMS stage during the 21 days (Figure 6b,c).

### 3.4. Effects of Chronic Administration of GT on Delta Power of NREMS

**Figure 7:**
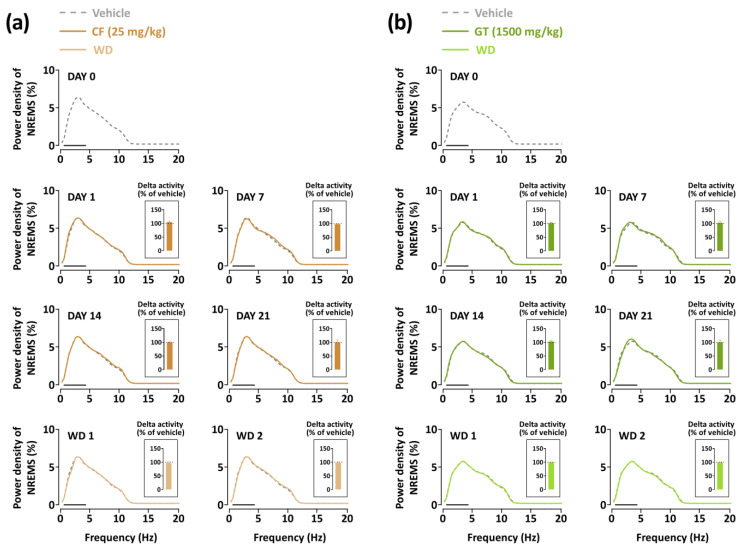
Changes in the EEG power density curve in NREMS during the administration of (**a**) caffeine and (**b**) GT. Grey curves illustrate the baseline day (vehicle). Brown and green curves illustrate caffeine and GT treatments, respectively. Light brown and green curves illustrate the withdrawal (WD) days. The solid bar (—) illustrates the delta wave range from 0.5 to 4 Hz. The delta activity compared with vehicle is presented in the inset bar graph. CF, caffeine; GT, green tea ethanol extract; NREMS, non-rapid-eye-movement sleep; Wake, wakefulness.

The evaluation of sleep quality was conducted through EEG power density measurements during NREMS. The delta activity was observed in the frequency range of 0.5–4 Hz. In the caffeine-administered group, a change in EEG power density was not induced for the three weeks compared to the baseline group (Figure 7a). When the caffeine treatment was discontinued, the power density of NREMS was maintained at a normal level. GT treatment did not alter the delta activity in NREMS throughout the three weeks, similar to that of the vehicle (Figure 7b). These results suggest that GT and caffeine have lasting arousal-inducing effects without any adverse effects or tolerance phenomena.

### 3.5. Caffeine Content of GT

**Figure 8:**
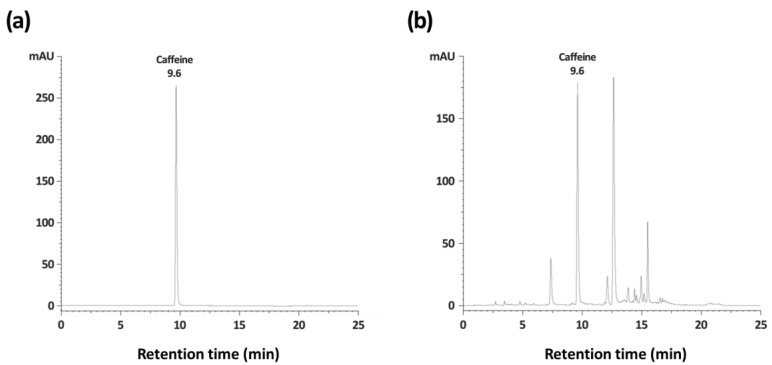
HPLC analysis chromatogram of caffeine standard (**a**) and GT (**b**). GT, green tea ethanol extract.

For comparison with caffeine at 25 mg/kg, which was used as a positive control, we analyzed the caffeine content of GT using an HPLC system. The retention time of caffeine was 9.6 min, and the caffeine content of GT was 63.9 ± 1.9 mg/g (Figure 8). Compared to the positive control, GT at 1500 mg/k had more caffeine content (95.6 mg/kg) than at 25 mg/kg. This result may suggest that other components of GT affected the arousal-inducing effect of GT.

## 4. Discussion

Caffeine, one of the most popular food constituents globally, is usually consumed as a diverse beverages to help induce awakening [27]. Previous studies established that moderating caffeine intake did not cause harmful effects, such as anxiety, depressive symptoms, and cognitive failures [28], and has beneficial effects associated with elevation of alertness, attention, and cognitive function [29]. A previous study by Huang et al. [7] reported a significant increase in the wakefulness of caffeine-treated mice at a dose of 15 mg/kg. In addition, several clinical studies have identified the maintenance of wakefulness by caffeine [30,31]. As expected, our findings revealed that caffeine, used as a positive control, significantly increased sleep latency and maintained arousal effects in wakefulness during the 3 h after treatment (Figure 2a and Figure 3).

In this study, we identified, for the first time, that the administration of GT presented arousal-inducing effects in mice throughout the treatment period. Compared to caffeine, GT treatment also significantly delayed sleep latency and elevated wakefulness in the 3 h following administration. In the analysis of sleep–wake episodes, GT produced a significant decrease in the number of stage transitions from wakefulness to NREMS and from NREMS to wakefulness (Figure 6a). Given that the effects were similar to those of caffeine, these results imply that GT maintained wakefulness without frequent shifts. The administration of caffeine significantly decreased the number of bouts of wakefulness ranging from 10 to 30 s during the 21 days (Figure 6b). However, a previous study reported that a significant decrease was not induced in the number of bouts of wakefulness ranging from 10 to 30 s [11]. Because we investigated the sleep-promoting effects of extract using the caffeine-induced sleep disruption model, our study was performed to evaluate the arousal-inducing effect of GT. Thus, it was administered at different times to confirm the opposite effects of samples. According to a study by Cao et al. [32] study, daytime administration preferable, corresponding to the resting phase in rodents.

Our findings suggest that the arousal-inducing effects of GT may be induced primarily by caffeine, the main compound responsible for wakefulness in green tea [33]. The U.S. Food and Drug Administration recommends a daily caffeine intake of up to 400 mg/day, which is equivalent to approximately four to five cups of coffee [34]. When converted to caffeine content of GT of 1500 mg/kg, compared to recommended dosage, it is less than a quarter of the amount. In addition, a commonly consumed coffee contains 80 to 100 mg of caffeine per 8 oz (240 mL). However, GT (caffeine content of 1500 mg/kg–about 95.6 mg/kg) contains about four times more caffeine than the dose used as the positive control (25 mg/kg) in this experiment, although the arousal-inducing effect was not higher than that reported in the positive control. These results imply that other constituents of GT inhibit the arousal effect of caffeine.

EGCG, which is known to induce hypnotic effects, was contained in dried green tea leaves in concentrations ranging from 8 to 12% [35]. In consecutive studies by Park et al. [36,37], EGCG augmented sleep-promoting effects and counteracted caffeine-induced anxiogenic effects in mice. L-theanine in concentrations of between 1% and 2% in dry weight of green tea is known to relieve stress and reduce anxiety [38]. Additionally, a previous study reported that L-theanine recovered slow-wave sleep to normal levels in caffeine-induced sleep disturbance [39]. Although green tea contains small amounts, quercetin and kaempferol have also been studied for their sleep-promoting effects. Kambe et al. [40] found that quercetin at a dose of 200 mg/kg significantly elevated NREMS in rats. Kaempferol also potentiated sleep-promoting effects in the pentobarbital-induced sleep test [41]. In view of these results, GT may be a better alternative for mild arousal-inducing effects than consumption of caffeine alone. Figure 6c supports that caffeine reduced the number of bouts of the NREMS stage lasting longer than 30 s during the first 3 h, but GT had no effect during the NREMS stage.

Similar to a previous study [42], our research clearly demonstrates that administration of caffeine causes a significant increase in wakefulness for up to 5 h (Figure 3). On the other hand, the administration of GT promoted weaker arousal-inducing effects than those promoted by the consumption of caffeine (Figure 4). As evident from previous studies, other constituents of GT with sleep-promoting effects could downregulate the arousal-inducing effects. The delta activity is used as a sleep parameter to evaluate sleep intensity in NREMS [43,44]. This study confirmed that caffeine did not change the delta activity [11] and that caffeine, as well as GT, induces arousal-inducing effects without a change in the delta activity. To evaluate whether GT presents withdrawal symptoms or adverse effects using EEG and EMG, recordings in withdrawal days were analyzed in the mice. As we previously reported [45], caffeine did not induce a tolerance phenomenon or withdrawal symptoms after treatment completion (Figure 7a). In addition, it was demonstrated that GT clearly induced arousing effects without any tolerance phenomenon over the three weeks or withdrawal symptoms after the end of treatment (Figure 7b). This study suggests that chronic administration of GT maintains arousal-inducing effects without any side effects.

## 5. Conclusions

The finding of the present study reveal that, similar to caffeine, GT maintained arousal-inducing effects during the chronic administration period. As far as we know, this study is the first investigation to elucidate the arousal-inducing effect of GT through its chronic administration in mice. However, it is necessary to further investigate which other components of GT may cause arousal-inducing effects and how they correlate with caffeine. Collectively, this study indicates that GT may be a promising stimulant without any withdrawal symptoms. Furthermore, the arousal-inducing effects of GT without caffeine to prevent these effects should be considered prior to using GT as a dietary supplement. Considering the diverse effects of GT, it may be available as a multifunctional ingredient.

## Data Availability

Not applicable.
